# The immune factors have complex causal regulation effects on kidney stone disease: a mendelian randomization study

**DOI:** 10.1186/s12865-024-00627-x

**Published:** 2024-06-14

**Authors:** Dongfeng Yuan, Junyi Yang, Weisong Wu, Yirixiatijiang Amier, Xianmiu Li, Wenlong Wan, Yisheng Huang, Jiabo Li, Xiao Yu

**Affiliations:** 1 Department of Urology, Institute of Urology, Tongji Hospital, Tongji Medical College, Huazhong University of Science and Technology, Wuhan, 430030 China; 2 Department of Urology, Tongji Hospital, Tongji Medical College, Huazhong University of Science and Technology, Liberalization Ave, No. 1095, Wuhan, 430030 China

**Keywords:** Immune cell, Kidney stone disease, Causal relationship, Mendelian randomization, GWAS

## Abstract

**Purpose:**

Previous studies have reported the potential impact of immune cells on kidney stone disease (KSD), but definitive causal relationships have yet to be established. The purpose of this paper is to elucidate the potential causal association between immune cells and KSD by Mendelian randomization (MR) analysis.

**Methods:**

In our study, a thorough two-sample Mendelian randomization (MR) analysis was performed by us to determine the potential causal relationship between immune cell traits and kidney stone disease. We included a total of four immune traits (median fluorescence intensity (MFI), relative cellular (RC), absolute cellular (AC), and morphological parameters (MP)), which are publicly available data. GWAS summary data related to KSD (9713 cases and 366,693 controls) were obtained from the FinnGen consortium. The primary MR analysis method was Inverse variance weighted. Cochran’s Q test, MR Egger, and MR-Pleiotropy RESidual Sum and Outlier (MR-PRESSO) were used to assess the stability of the results.

**Results:**

After FDR correction, the CD8 on HLA DR + CD8br (OR = 0.95, 95% CI = 0.93–0.98, p-value = 7.20 × 10^− 4^, q-value = 0.088) was determined to be distinctly associated with KSD, and we also found other 25 suggestive associations between immune cells and KSD, of which 13 associations were suggested as protective factors and 12 associations were suggested as risk factors. There was no horizontal pleiotropy or significant heterogeneity in our MR analysis, as determined by the p-value results of our Cochrane Q-test, MR Egger’s intercept test, and MR-PRESSO, which were all > 0.05.

**Conclusions:**

Our study has explored the potential causal connection between immune cells and KSD by Mendelian randomization analysis, thus providing some insights for future clinical studies.

**Supplementary Information:**

The online version contains supplementary material available at 10.1186/s12865-024-00627-x.

## Introduction

Kidney stone disease (KSD) is a common and frequent disease whose incidence is steadily increasing worldwide. It has been found that the prevalence of KSD in the United States is about 10% [1]. Not only that, the prevalence and recurrence of kidney stones in children are gradually increasing worldwide [2]. KSD has a high rate of recurrence, with approximately 50% recurrence within 5–10 years [3]. KSD is prone to a variety of complications, including pain, urinary tract obstruction, infection, and even increases the likelihood of chronic kidney disease, which may ultimately develop into end-stage renal disease [4, 5]. However, the risk factors influencing the development of KSD have not been fully investigated. Treatments such as medications and minimally invasive surgery can only remove formed stones and have little effect on reducing recurrence or early prevention of kidney stones. Previous studies have found a correlation between some immune cells and the development of KSD [6]. Paul R. Dominguez-Gutierrez et al. suggest that by modulating the immune response, immunotherapy may offer a way to prevent stone recurrence in some people [7]. Immunotherapy essentially enhances cellular immunity, regulates the degree of immune activation, and improves immune cell function, thereby inhibiting the occurrence and development of various diseases [8]. Personalized neoantigen vaccine is a novel immunotherapeutic approach that uses long synthetic peptides to induce neoantigen-specific T-cell responses in patients. The peptides directly initiate the immune system and have the advantage of simple and rapid production [9]. It has been shown that peptide vaccines targeting neoantigens can effectively induce T-cell responses [10]. Then, it is worth considering and exploring whether we can use personalized neoantigen vaccines to modulate the body’s immune cell response to achieve early prevention and treatment of KSD. Therefore, it is particularly significant to explore the role played by immune cells in the formation of kidney stones. However, due to the difficulties of observational studies and the limitations of analyzing the results, evidence of a potential association between immune cells and KSD is not readily available through this method and the results are prone to reverse causation. Thus, the potential causal relationship between immune cells and KSD needs to be explained by more precise research methods. Mendelian randomization (MR) is a genetic epidemiological method that uses genetic variants as instrumental variables (IVs) to assess the causality between risk factors and target diseases [11]. MR utilizes random assignment of single nucleotide polymorphisms (SNPs) in genetic variants to simulate randomized trials in populations, thus overcoming potential confounders and interferences of reverse causality [12]. It is very critical that the causal sequence of MR be rational [13, 14]. Therefore, we obtained summary statistics from large genome-wide association studies (GWAS) and revealed the causal relationship between immune cells and KSD by a two-sample MR analysis.

## Materials and methods

### Study design

**Fig. 1:**
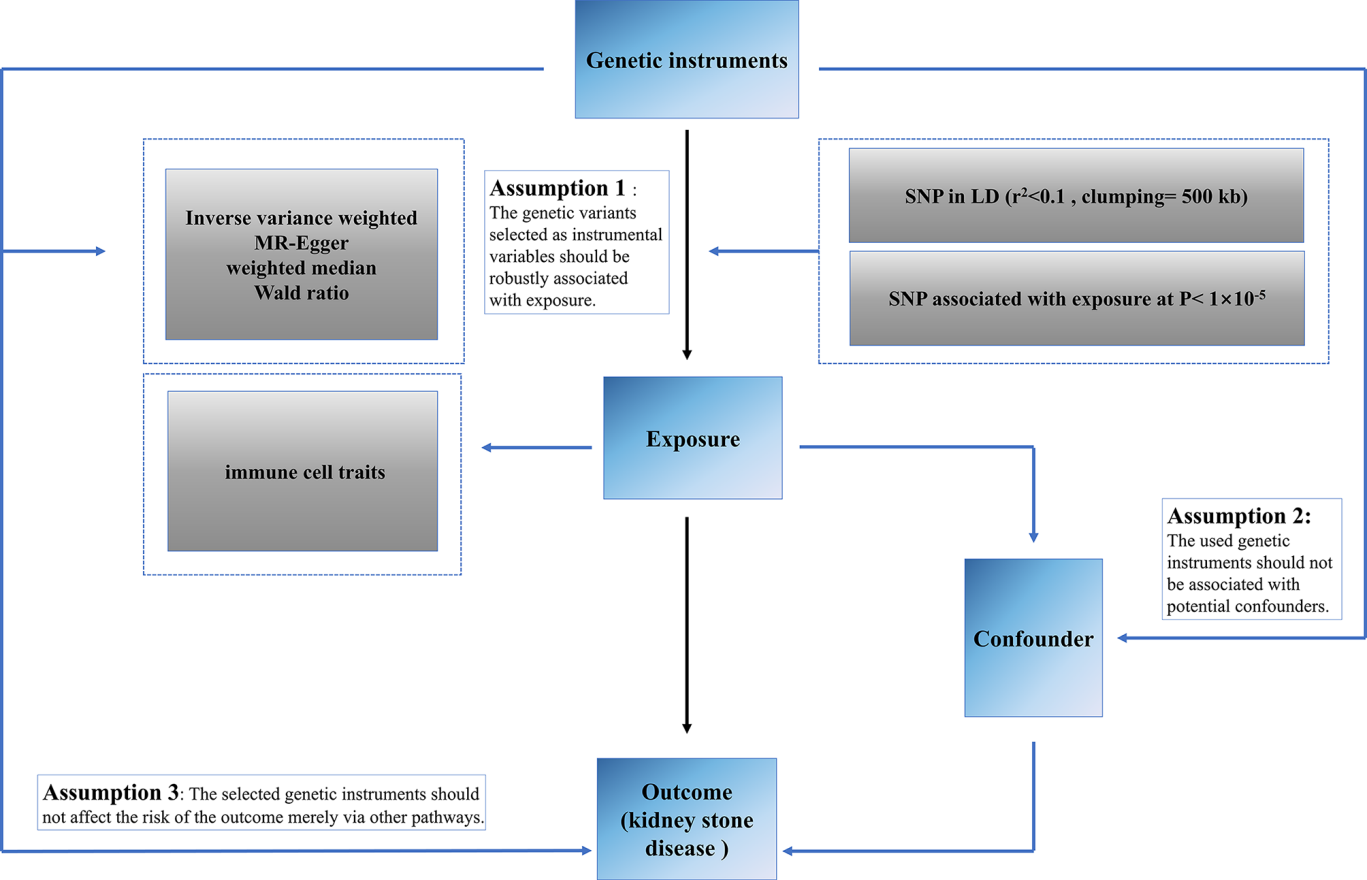
Overview and assumptions of the Mendelian randomization study design. Significant instrumental variables were selected for immune cells and KSD, and the causalities were then explored. Three basic assumptions of MR analysis were illustrated in this graph, namely, relevance (assumption 1), independence (assumption 2), and exclusion restrictions (assumption 3)

Our study followed the STROBE-MR statement used to report MR research [15]. No additional ethical approvals were required for this study, and the data we used and analyzed were published and publicly available data. An overview of the study design is shown in Fig. 1.

### Genetic instrument selection

A summary of GWAS statistics for each immune trait is publicly available from the GWAS Catalog (accession numbers from GCST0001391 to GCST0002121) [16]. We included immunophenotypes that belonged to four different immune traits: absolute cell (AC) count, median fluorescence intensity (MFI), morphologic parameter (MP), and relative cell (RC) count. When categorized by cell panels, they belong to seven different panels, the Treg, TBNK (T cells, B cells, natural killer cells), B cells, monocytes, CDCs, myeloid cells, and mature stages of T cells. Initial immune traits GWAS was obtained from data on 3,757 Europeans and there were no overlapping cohorts. Approximately 22 million SNPs genotyped with high-density arrays were estimated using a reference panel based on Sardinian sequences and tested for correlation after adjusting for covariates [17]. 

According to recent research [16, 18], we set the significance level of IVs for each immune trait at 1 × 10^− 5^. Meanwhile, all SNPs were clumped according to linkage disequilibrium (LD), defined by r ^2^ < 0.1 with a clumping window > 500kb [19]. In this way, we could include more SNPs to ensure the stability of the results. We removed palindromic SNPs as well as SNPs that could not be found in the KSD data.

### Data source for kidney stone disease

We obtained research data on kidney stone disease (KSD) from the FinnGen consortium, derived from the ninth release of the FinnGen consortium with 9,713 cases and 366,693 controls of kidney stone disease.

### Statistical analysis

We first harmonized single nucleotide polymorphisms (SNPs) with the same allele, and then we performed two-sample MR analyses using inverse variance weighting (IVW), MR-Egger, and weighted median. The results primarily relied on the IVW method, with support from the other two methods. The MR-Egger method has the ability to tolerate potential pleiotropy and estimate results conservatively [20]. The weighted median method is a method that provides reliable estimates of causal effects and has the great advantage of allowing for 50% invalid IVs [21]. Also, we utilized the Wald ratio method to infer the effect of a single IV on KSD. We grouped the results of the IVW analysis according to the seven different cell panels, performed the false discovery rate (FDR) correction according to the groups, and compared the corrected p-value, also known as q-value, with the threshold we set at 0.1 [22]. We considered the association between immune cells and KSD to be suggestive if the p-value was < 0.05 but the q-value was ≥ 0.1, and significant if the q-value was < 0.1. We used Cochrane’s Q-test to assess heterogeneity, along with the MR-Egger intercept to assess pleiotropy. To assess whether the results were affected by outlier SNPs, we used leave-one-out analysis, MR-Pleiotropy Residual Sum and Outlier method (MR-PRESSO) to validate. We excluded results with heterogeneity and horizontal pleiotropy and retained results with the same direction under the three analyses of IVW, MR-Egger, and weighted median. All analyses were performed with the software packages TwoSampleMR (version 0.5.7) and MR-PRESSO (version 1.0) in R (version 4.3.0), and the correlation heat map was drawn using ChiPlot (https://www.chiplot.online/**).**

## Results

### Overview

Table 1 lists all the results that reached a suggestive significance (p-value < 0.05) for a total of 26 pairs between immune traits and KSD. When categorizing results based on the cell panels,12 traits belonged to the Treg, 4 from TBNK, 3 classified as monocyte, 1 from Maturation stages of T cell, 2 belonged to cDC, and 4 within B cell were found to be suggestively associated with KSD. We corrected the p-values in the different panels using the FDR method to reduce possible false positive results (Supplementary Table S1). After sensitivity analysis and multiple correction analysis (Supplementary Table S2), the CD8 on HLA DR + CD8br remained statistically significant (q-value < 0.1). Meanwhile, we categorized immune cells into seven sections according to the panel, grouped complex findings according to the panel, and found multiple suggestive associations between immune cells and KSD.

**Table 1:** The results of the MR analysis between the 26 immune traits that reached suggestive significance and the risk of KSD

Exposure	Cell panel	Trait type	Outcome	Method	NO. of SNP	β	*p*-value		OR (95% CI)	q-value	
CD28 on CD39 + CD4+	Treg	MFI	KSD	IVW	53	-0.039	3.70E-03		0.96 (0.94–0.99)	0.310	
CD39 + CD4 + AC	Treg	Absolute count	KSD	IVW	27	-0.033	7.60E-03		0.97 (0.94–0.99)	0.422	
CD25 + + CD8br %CD8br	Treg	Relative count	KSD	IVW	20	-0.048	0.027		0.95 (0.91–0.99)	0.735	
CD39 + secreting Treg %CD4 Treg	Treg	Relative count	KSD	IVW	156	-0.015	0.030		0.99 (0.97-1.00)	0.618	
CD25 on CD39 + CD4+	Treg	MFI	KSD	IVW	76	0.021	0.032		1.02 (1.00-1.04)	0.596	
CD39 + secreting Treg %secreting Treg	Treg	Relative count	KSD	IVW	164	-0.014	0.038		0.99 (0.97-1.00)	0.527	
CD28 + CD45RA + CD8dim %T cell	Treg	Relative count	KSD	IVW	53	0.013	0.038		1.01 (1.00-1.03)	0.578	
CD28 + CD45RA + CD8br %T cell	Treg	Relative count	KSD	IVW	62	0.006	0.042		1.01 (1.00-1.01)	0.495	
CD28- CD25 + + CD8br AC	Treg	Absolute count	KSD	IVW	20	0.030	0.042		1.03 (1.00-1.06)	0.537	
CD39 on CD39 + secreting Treg	Treg	MFI	KSD	IVW	130	-0.014	0.043		0.99 (0.97-1.00)	0.480	
CD3 on CD39 + CD4+	Treg	MFI	KSD	IVW	66	0.019	0.044		1.02 (1.00-1.04)	0.455	
CD28- CD25 + + CD8br %T cell	Treg	Relative count	KSD	IVW	27	0.050	0.049		1.05 (1.00-1.11)	0.477	
CD8 on HLA DR + CD8br	TBNK	MFI	KSD	IVW	29	-0.049	**7.20E-04**		0.95 (0.93–0.98)	**0.088**	
CD8br NKT %lymphocyte	TBNK	Relative count	KSD	IVW	27	0.086	2.90E-03		1.09 (1.03–1.15)	0.180	
HLA DR on HLA DR + CD8br	TBNK	MFI	KSD	IVW	18	-0.065	0.017		0.94 (0.89–0.99)	0.353	
CD3- lymphocyte %leukocyte	TBNK	Relative count	KSD	IVW	18	0.046	0.031		1.05 (1.00-1.09)	0.548	
CX3CR1 on CD14 + CD16 + monocyte	Monocyte	MFI	KSD	IVW	45	-0.031	0.015		0.97 (0.95–0.99)	0.612	
CX3CR1 on CD14 + CD16- monocyte	Monocyte	MFI	KSD	IVW	42	-0.030	0.035		0.97 (0.94-1.00)	0.494	
CD64 on CD14 + CD16- monocyte	Monocyte	MFI	KSD	IVW	83	0.019	0.040		1.02 (1.00-1.04)	0.422	
TD DN (CD4-CD8-) %DN	Maturation stages of T cell	Relative count	KSD	IVW	30	-0.012	0.031		0.99 (0.98-1.00)	1.226	
CCR2 on monocyte	cDC	MFI	KSD	IVW	36	0.044	2.50E-03		1.04 (1.02–1.07)	0.157	
CCR2 on CD62L + myeloid DC	cDC	MFI	KSD	IVW	24	0.048	9.50E-03		1.05 (1.01–1.09)	0.301	
IgD- CD38br %B cell	B cell	Relative count	KSD	IVW	12	-0.082	8.30E-03		0.92 (0.87–0.98)	0.142	
CD27 on CD24 + CD27+	B cell	MFI	KSD	IVW	73	0.019	0.025		1.02 (1.00-1.04)	0.278	
IgD- CD27- %lymphocyte	B cell	Relative count	KSD	IVW	18	-0.056	0.026		0.95 (0.90–0.99)	0.274	
CD20 on memory B cell	B cell	MFI	KSD	IVW	46	-0.041	0.038		0.96 (0.92-1.00)	0.375	

MR, Mendelian randomization; SNP, single nucleotide polymorphism; OR, odds ratio; CI, confidence interval; IVW, inverse variance weighted.

### Treg and KSD

**Fig. 2:**
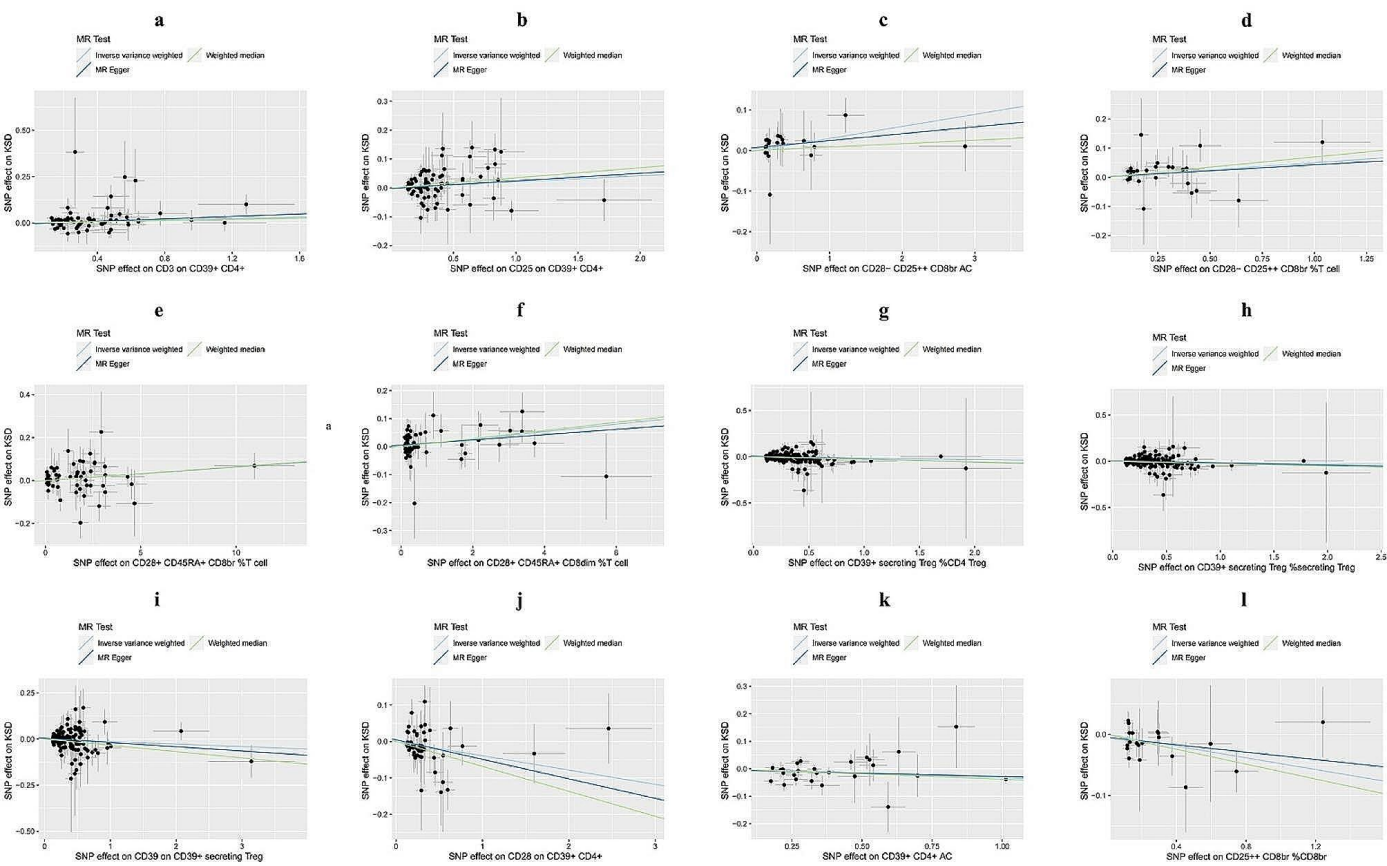
Scatter plot for the relationship between the SNP effect size of causal immune traits (x-axis) and the corresponding effect size estimates of kidney stone disease (KSD) (y-axis). The a ~ l plots all belong to the Treg group panel. (a) CD3 on CD39 + CD4 + on KSD, (b) CD25 on CD39 + CD4 + on KSD, (c) CD28- CD25 + + CD8br AC on KSD, (d) CD28- CD25 + + CD8br %T cell on KSD, (e) CD28 + CD45RA + CD8br %T cell on KSD, (f) CD28 + CD45RA + CD8dim %T cell on KSD, (g) CD39 + secreting Treg %CD4 Treg on KSD, (h) CD39 + secreting Treg %secreting Treg on KSD, (i) CD39 on CD39 + secreting Treg on KSD, (j) CD28 on CD39 + CD4 + on KSD, (k) CD39 + CD4 + AC on KSD, (l) CD25 + + CD8br %CD8br on KSD.

12 pairs between Treg/KSD reached suggestive association (p-value < 0.05) by using IVW MR analysis. RC had the greatest number of suggestive associations than other trait types. CD39 and CD28 were the most commonly expressed molecules in different types of immune traits. And, CD28 on CD39 + CD4 + was the most essential trait compared with other traits in this section (p-value = 3.70 × 10^− 3^). Furthermore, as shown in the scatter plot (Fig. 2), the traits (e.g., CD3 on CD39 + CD4+, CD25 on CD39 + CD4+) were positively associated with KSD (Fig. 2a–f), while the traits (e.g., CD39 + secreting Treg %CD4 Treg, CD39 + secreting Treg %secreting Treg) were negatively associated with KSD (Fig. 2g-l).

### TBNK and KSD

**Fig. 3:**
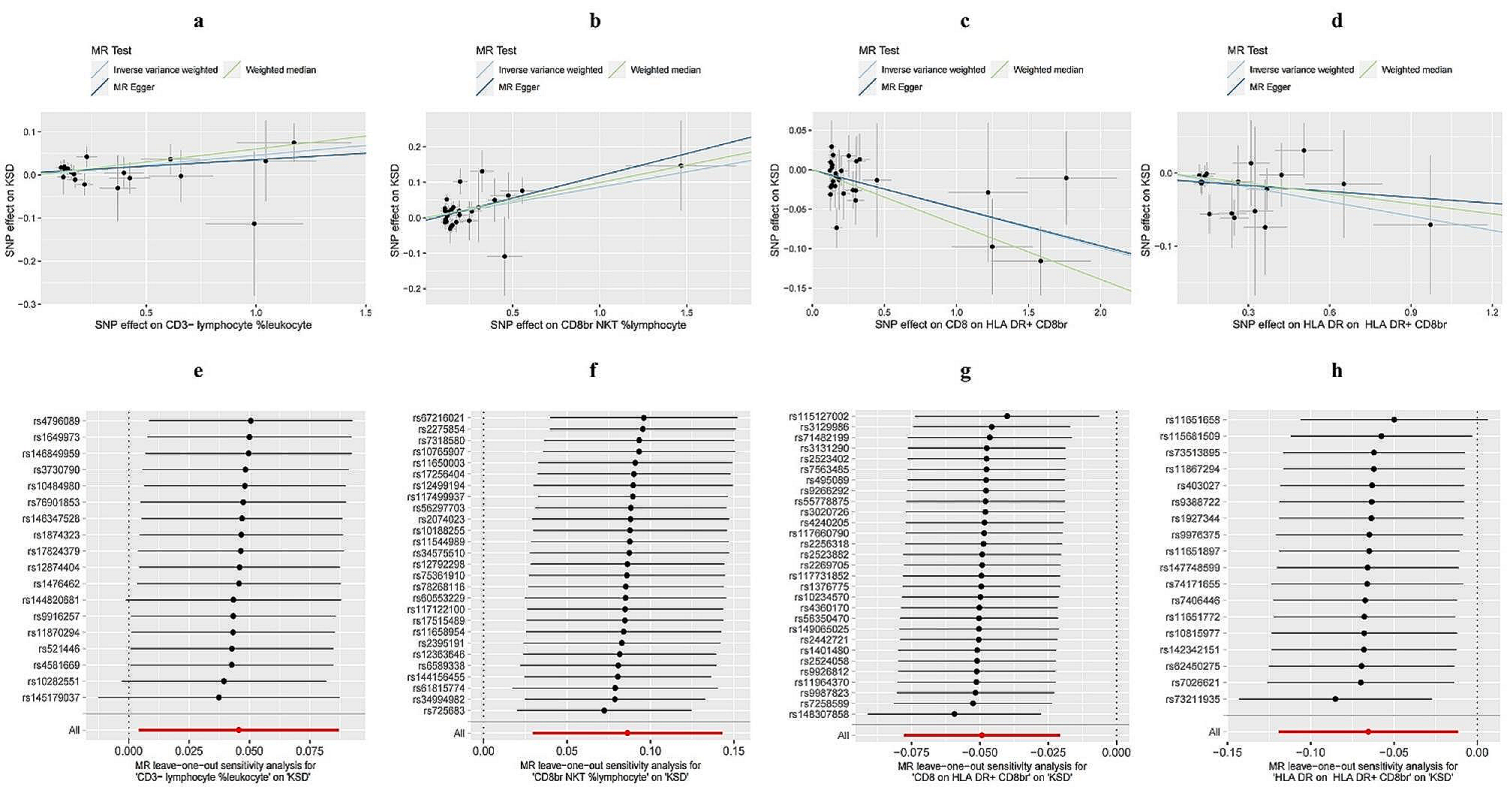
Scatter plot for the relationship between the SNP effect size of causal immune traits (x-axis) and the corresponding effect size estimates of kidney stone disease (KSD) (y-axis) and leave-one-out plot for the causal relationships between the TBNK group panel and KSD. The a ~ d scatter plots and e ~ h leave-one-out plots all belong to the TBNK group panel. (a) and (e) CD3- lymphocyte %leukocyte on KSD, (b) and (f) CD8br NKT %lymphocyte on KSD, (c) and (g) CD8 on HLA DR + CD8br on KSD, (d) and (h) HLA DR on HLA DR + CD8br on KSD.

**Fig. 4:**
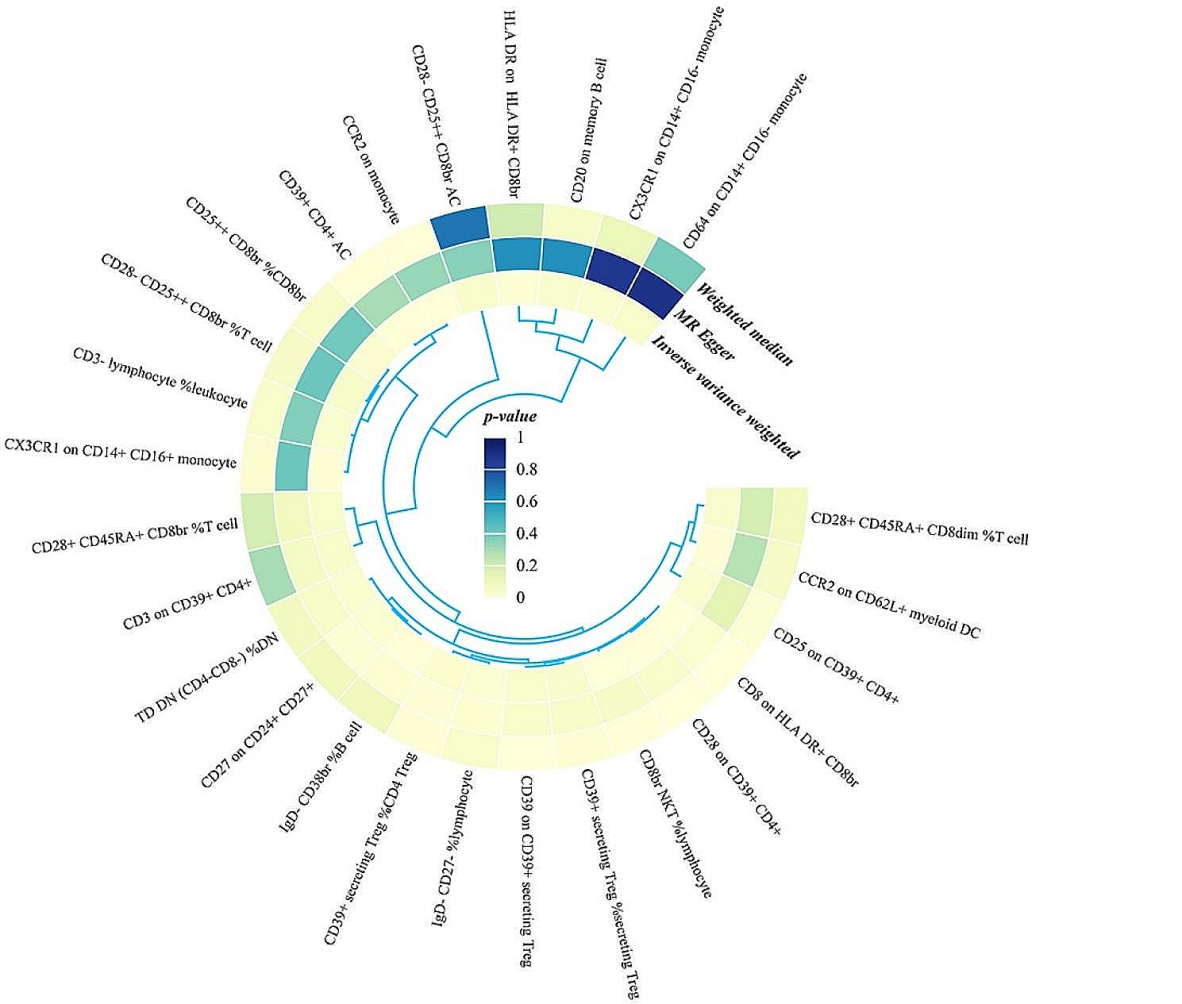
Circle heatmap of the results of the MR analysis between the 26 immune traits that reached suggestive significance and the risk of KSD. The circle from the outer to the inner represented the weighted median, MR-Egger, and IVW, respectively

Table 1 shows the suggestive significance of the 4 pairs between the TBNK panel and the KSD (p-value < 0.05). RC and MFI had the largest number of suggestive associations. After FDR correction, the CD8 on HLA DR + CD8br (OR = 0.95, 95% CI = 0.93–0.98, p-value = 7.20 × 10^− 4^, q-value = 0.088) had significant causal effects on KSD estimated from IVW (q-value < 0.1) (Supplementary Table S1). Moreover, for the most significant pair, no significant heterogeneity or horizontal pleiotropy was detected by sensitivity analysis (Supplementary Table S2), and the leave-one-out plot showed robust results for the relationship between CD8 on HLA DR + CD8br and KSD (Fig. 3g). We observed consistent correlation signals using other methods (Fig. 4) (Supplementary Table S3). As shown in Fig. 3, negative associations were found between CD8 on HLA DR + CD8br, HLA DR on HLA DR + CD8br, and KSD. On the other hand, the positive associations between CD3- lymphocyte %leukocyte, CD8br NKT %lymphocyte, and KSD were also detected. (Fig. 3a-d)

### B cell, Monocyte, cDC, and KSD

A total of 4 B cell /KSD pairs, 3 Monocyte/KSD, and 2 cDC/KSD were detected at the suggestive level of p-value < 0.05(Table 1). Among them, the traits of MFI were particularly prominent, as a high percentage of suggestive significant pairs was detected up to 77.78% (7 MFI pairs vs. 9 total pairs). Meanwhile, 13 of the 26 pairs of results obtained were classified as belonging to MFI based on immune traits, which further suggested that immune cells belonging to MFI may play an important role in the development of KSD. It should be noted that BAFF-R on sw mem, which belongs to the B cell panel, was significantly associated with KSD after correction FDR correction (q-value = 0.099) (Supplementary Table S1). However, after the Cochrane Q-test and MR Egger’s intercept test, the results were of heterogeneity and pleiotropy, so we did not include it in the discussion.

### Maturation stages of T cell, and KSD

Compared to the 12 pairs of results from Treg and KSD, only 1 pair between the Maturation stages of T cell /KSD reached suggestive association (p-value < 0.05) (Table 1). This suggests that Treg may have a more complex role in the progression of KSD compared to the Maturation stages of T cell.

### Myeloid cell

Although five immune cell trait types in the Myeloid cell panel showed suggestive correlation with KSD, and one of them, CD33 on CD33dim HLA DR-, remained significantly correlated after FDR correction (Supplementary Table S1). Nevertheless, after the Cochrane Q-test and MR Egger’s intercept test, the results were of heterogeneity and pleiotropy, so we excluded the results in the Myeloid cell panel from the discussion.

### Sensitive analysis

Cochran’s Q test, MR-Egger intercept test, and MR-PRESSO showed no heterogeneity or horizontal pleiotropy in the 26 pairs of results we retained in the analyses of immune cells and risk of KSD (all p-value > 0.05) (Supplementary Table S2). The leave-one-out plot and scatter plot showed that the results were robust.

## Discussion

Despite the important role of the immune system in the formation of KSD, current research remains unclear as to its specific complex role in the progression of KSD. As far as we know, it’s the first time that the potential causal relationship between multiple types of immune cells and KSD has been explored by MR analytical methods. Our study found 12, 4, 4, 3, 2, 1, pairs between 6 panels of immune traits (i.e., Treg, TBNK, B cell, Monocyte, cDC, Maturation stages of T cell) and KSD were detected at the suggestive level of p-value < 0.05, respectively. Among them, the CD8 on HLA DR + CD8br/KSD reached the level of significant correlation (q-value < 0.1).

We found that the risk of KSD decreased as the proportion of the CD8 on HLA DR + CD8br increased. In the TBNK panel, T lymphocytes were split into six subsets based on the expression of the CD4 and CD8 markers: CD4-CD8- (DN), CD4-CD8dim (CD8dim), CD4-CD8bright (CD8br), CD4 + CD8br (DP), CD4 + CD8dim, CD4 + CD8- (CD4+) [16]. The HLA DR positivity of CD4 + and CD8br T cells and NK cells was considered as an activation marker [16].

CD8 on HLA DR + CD8br, which can be interpreted as a CD4-CD8bright (CD8br) subset of T lymphocytes in the HLA-DR activated state. CD8 is a heterodimer composed of α and β peptide chains, and its main function is to assist in the process of antigen recognition by immune cells and to participate in the activation of T cells. signals, which can enhance the interaction between T-cells and APCs or target cells [23]. CD8br cells are limited by their own MHC class I molecules and differentiate into cytotoxic T cells (CTLs) with cytotoxicity after activation, which can specifically kill target cells. Antigens or cytokines activate these cells, and activated cells induce apoptosis through two primary pathways: lytic (cytotoxicity) and non-lytic (cytokine production) mechanisms respectively [24]. The lysis process mainly utilizes secreted particles containing effector substances, such as granzyme, perforin, etc. These particles are released to act directly on the target cells, thereby killing them. Non-lytic mechanisms mainly refer to the secretion of cytokines such as IFN-γ and TNF-α, which are essential for inflammatory responses [25]. Meanwhile, CD8 + T cells in infectious diseases have been reported to produce cytokines to act on themselves, thereby enhancing their cytotoxic activity upon activation [26, 27].

An association between kidney stone formation and urinary tract infections has been reported in certain patient groups [28, 29]. Localized infection in the urinary tract is one of the factors that trigger stone formation, and the stones themselves are foreign bodies that can cause obstruction in the urinary tract, which can exacerbate the degree of infection, and then enter a vicious circle [30]. Urease-producing bacteria such as Proteus mirabilis, Klebsiella pneumoniae, Staphylococcus aureus, and Pseudomonas aeruginosa are invariably associated with the formation and recurrence of struvite calculi [31]. These bacteria split urea and promote the formation of ammonia and carbon dioxide, leading to renal tubular damage and alkalinization of the urine with subsequent formation of phosphate and struvite calculi [31]. Also, de Cógáin et al. described secondary infection stones caused by non-urease-producing bacteria, including Escherichia coli and Enterococcus spp [32, 33]. During the formation of kidney stones, cytotoxic T cells are able to directly kill the bacteria that cause urinary tract infections. They do this by releasing cytotoxins, such as granzymes and perforins, which disrupt the cell membranes of the pathogens, thereby triggering the death of the bacteria. This direct killing effect can effectively control and clear the infection, preventing it from spreading further and leading to the deterioration of kidney stones. Using immunoinformatics and structural vaccinology, Suar et al. designed multi-epitope peptide vaccines against Klebsiella pneumoniae [34], Enterococcus faecalis [35], Staphylococcus aureus [36, 37], and Acinetobacter baumannii [38, 39] with highly immunogenic cytotoxic T-lymphocyte epitopes and high binding affinities for most human leukocyte antigen (HLA). This offers feasibility and credibility for our future utilization of vaccines to modulate immune responses, such as T-cell activation, to target bacteria during kidney stone formation and thereby prevent KSD.

In the early stages of kidney stone formation, the primary role of CD8 + T cells is to activate and direct the involvement of other immune cells in the immune response. They are able to secrete a range of cytokines, such as IFN-γ, and TNF-α, to promote an inflammatory response. Cytokines are a class of proteins that regulate the function of immune cells and participate in inflammatory responses and cell proliferation [40], and they play an important role in kidney stone formation [6]. TNF-α, upon stone stimulation of renal tubular epithelial cells, leads to aggregation of immune cells and induces an inflammatory response [6], thereby further damaging renal tissue. CD8 + T cells are also able to interact with other types of immune cells, such as macrophages and B-cells, to regulate the extent and direction of the immune response through the mediation of cytokines [41]. This immunomodulatory effect can limit excessive inflammatory responses, reduce kidney stone damage to kidney tissue, and aid in stone expulsion and removal. These findings are consistent with those of Noble et al. They found that CD8 + T cells can secrete anti-inflammatory cytokines with inhibitory activity, such as IL-10 [42]. Anti-inflammatory cytokines (e.g., IL-10) inhibit the inflammatory response, while it has been suggested that exogenous IL-10 treatment can reverse oxalate-mediated outcomes in vitro [43, 44]. Meanwhile, IL-10 was identified as a urinary tract inflammation-associated factor that can accurately differentiate between control individuals and patients with urinary tract stones [45]. Equally important, IL-10 has been reported to be essential for the induction of M2 macrophage polarization [46]. M2 anti-inflammatory macrophages have been found to phagocytose and degrade CaOx kidney stone fragments [47]. Because the M2 phenotype is more able to phagocytose crystals than the M1 phenotype [48], regulating the direction of their polarization may have therapeutic value [49]. Taguchi, K et al. found that M1Mϕ infusion and LPS and IFN-γ induction promoted renal crystal formation, whereas M2Mϕ infusion and IL-4 and IL-13 induction inhibited renal crystal formation [48]. Zhu et al. also modulated macrophage CSF-1 expression by inhibiting androgen receptor, thereby reducing crystal deposition in the kidney by altering macrophage recruitment/M2 polarization [50]. These experimental pieces of evidence provide us with the possibility of utilizing the cytokines secreted by CD8 + T cells to regulate the polarization of macrophages for the treatment of kidney stones. The combination of effective molecular adjuvants can cause massive expansion of antigen-specific CD8 T cells and show previously unseen efficacy in vaccine and tumor models [51], so whether we can utilize them for the treatment of KSD deserves to be explored further in the future.

Our findings may suggest that immune cells have a complex role in the pathogenesis and progression of KSD. These new findings may contribute to a more comprehensive understanding of immune cells in KSD and provide new potential biomarkers for early diagnosis and monitoring of KSD. Meanwhile, these findings also raise the question of what role these immune cells could play in KSD prevention and treatment. Currently, the recurrence rate of KSD after surgery remains high due to individual differences and environmental factors [52], and the damage to the kidney from repeated surgeries can easily lead to life-threatening renal failure. In addition, there is a lack of effective drugs for the treatment of kidney stones. Then immunotherapy based on the results of our findings may provide new ideas for the prevention and treatment of kidney stones. The application of immunotherapy has revolutionized cancer treatment by harnessing the body’s immune system to work against cancer and significantly improve patient prognosis [53]. Based on our findings and what has been discussed above, we propose the following hypothesis for the application of immunotherapy against KSD: firstly, we can design a multiple-epitope peptide vaccine that induces a CD8 + T-cell immune response to directly kill bacteria in infected kidney stones. Secondly, the infusion of exogenous cytokines can be utilized to promote the polarization of M2 macrophages and reduce renal crystal deposition. Finally, combined vaccine molecular adjuvants can be designed to induce the expansion of CD8 + T cells. Then exploring the specific relationship between the interactions of these immune cells so as to accurately regulate the immune microenvironment of the renal mesenchyme and thus intervene in kidney stone formation by utilizing immunotherapeutic methods will be the focus of future research.

This study was the result of a two-sample MR analysis based on published results from a large genomic cohort study and was therefore statistically valid. The findings of this study were derived from causal inference utilizing various MR analysis methods with genetic instruments as variables. These results are reliable, robust, and not confounded by horizontal pleiotropy and other factors. However, there are still some limiting parts of our study. First, even though we performed multiple sensitivity analyses, we were unable to fully assess the horizontal pleiotropy effect. Second, the data in this study comes from the European population, so it is worth considering whether the results can be generalized to other races, which limits the generalizability of the application of the results. Third, the lack of individual-specific information prevented us from further stratifying the population. Finally, in order to be able to more fully explore the relationship between immune cells and kidney stones, we used a more relaxed threshold to assess the results, but this may have increased some false positives.

## Conclusion

In conclusion, we fully utilized the bidirectional MR analysis to investigate the potential relationship between immune cells and KSD, giving a little insight to researchers exploring the complex interactions between the immune system and KSD. And based on the results of the study, we propose hypotheses for future applications and future priorities that still need to be explored further. Moreover, our study reduces the potential for reverse causality as well as multiple unavoidable confounding factors in research, which may offer a novel approach for scientific researchers to continue exploring the Immunological mechanisms of KSD.

### Electronic supplementary material

Below is the link to the electronic supplementary material.

Supplementary Material 1

Supplementary Material 2

Supplementary Material 3

## Data Availability

The original contributions presented in this study are included in the article/Supplementary material, further inquiries can be directed to the corresponding author.

## Abbreviations

KSD: kidney stone disease
MFI: median fluorescence intensity
RC: relative cellular
AC: absolute cellular
MP: morphological parameters
GWAS: genome-wide association studies
IV: instrumental variable
IVW: inverse variance weighted
MR: Mendelian randomization
MR-PRESSO: MR-Pleiotropy Residual Sum and Outlier method
OR: odds ratio
SNP: single nucleotide polymorphism
CTLs: cytotoxic T cells
IL-10: Interleukin-10
IL-4: Interleukin-4
IL-13: Interleukin-13
IFN-γ: Interferon-gamma
LPS: Lipopolysaccharides
